# Profiling of tumour-associated microbiota in human hepatocellular carcinoma

**DOI:** 10.1038/s41598-021-89963-1

**Published:** 2021-05-19

**Authors:** Seiga Komiyama, Takahiro Yamada, Nobuyuki Takemura, Norihiro Kokudo, Koji Hase, Yuki I. Kawamura

**Affiliations:** 1 Division of Biochemistry, Faculty of Pharmacy and Graduate School of Pharmaceutical Science, Keio University, Tokyo, 105-8512 Japan; 2 Department of Surgery, National Center for Global Health and Medicine, Tokyo, 162-8655 Japan; 3 Department of Gastroenterology, Research Center for Hepatitis and Immunology, Research Institute, National Center for Global Health and Medicine, Chiba, 272-8516 Japan

**Keywords:** Hepatocellular carcinoma, Cellular microbiology

## Abstract

Liver cancer is the fourth leading cause of cancer-related death. Hepatocellular carcinoma (HCC) is a primary liver cancer that results from chronic hepatitis caused by multiple predisposing factors such as viral infection, alcohol consumption, and non-alcoholic fatty liver disease. Accumulating studies have indicated that dysfunction of the gut epithelial barrier and hepatic translocation of gut microbes may be implicated in the pathogenesis of HCC. However, the translocated bacteria in HCC patients remains unclear. Here, we characterised tumour-associated microbiota in patients with liver cancer and focused on HCC. We observed that the number of amplicon sequence variants in tumour-associated microbiota was significantly higher compared with that in non-tumour regions of the liver. The tumour-associated microbiota consisted of Bacteroidetes, Firmicutes, and Proteobacteria as the dominant phyla. We identified an unclassified genus that belonged to the *Bacteroides, Romboutsia,* uncultured bacterium of Lachnospiraceae as a signature taxon for primary liver cancer. Additionally, we identified *Ruminococcus gnavus* as a signature taxon for HCC patients infected with hepatitis B and/or hepatitis C viruses. This study suggests that tumour microbiota may contribute to the pathology of HCC.

## Introduction

Liver cancer is the fourth leading cause of cancer-related death worldwide (approximately 78,700 cases/year, 8.2% of all cancers)^1^. The prevalence of hepatocellular carcinoma (HCC), which accounts for more than 80% of primary liver cancers^2^, is associated with multiple environmental factors such as viral infection with hepatitis B virus (HBV) or hepatitis C virus (HCV), excessive alcohol consumption, and non-alcoholic fatty liver disease (NAFLD). These factors synergistically cause chronic inflammation in the liver, which eventually leads to HCC. In previous cohort studies, < 15% of HBV-infected Taiwanese participants had developed HCC within 13 years^3^, whereas the rate of HCC was 3% among HCV-infected individuals^2^. Similarly, the incidence rate of HCC was also at most 30% among patients with either alcohol-related liver disease or NAFLD^4^. These studies indicate that multiple rather than single predisposing factors are required to promote chronic inflammation.

Accumulating studies have suggested that gut epithelial barrier functions and microbiota are associated with the development of HCC^5,6^. In the intestines, a single layer of epithelial cells forms a physical barrier to prevent leakage of luminal contents including gut microbes into the body. Notably, patients with chronic liver diseases, such as alcoholic hepatitis, cirrhosis, and HCC, exhibit higher serum lipopolysaccharide (LPS) levels compared with healthy subjects, which indicates increased permeability of the gut epithelial barrier^7^. An animal study also demonstrated that chemical disruption of the epithelial barrier promotes tumorigenesis in the liver^8^. Thus, increased gut permeability has been implicated in tumorigenesis in patients with chronic liver diseases. This pathological event largely depends on Toll-like receptor 4 (TLR4) activation by LPS in hepatocytes, because genetic ablation of *Tlr4* suppresses HCC development in mice^9^. Moreover, epithelial barrier dysfunction facilitates translocation of certain bacteria into the liver. For example, mice transplanted with stool from alcohol hepatitis patients show translocation of cytolysin-positive *Enterococcus faecalis* from the gut to the liver upon administration of alcohol^10^. Detection of the cytolysin-positive *E. faecalis* strain is positively associated with mortality of alcohol hepatitis patients. These observations suggest that hepatic translocation of gut microbes is involved in the pathogenesis of other liver diseases such as HCC.

Recently, tumour tissues of several cancer types were found to be colonised with bacteria referred to as cancer microbiota, such as colorectal cancer and stomach cancer as well as tissues considered to be sterile, which included breast cancer and pancreatic ductal adenocarcinoma^11–13^. Cancer microbiota affects cancer pathology by regulating tumorigenesis, cancer progression, and resistance against chemotherapy^11,12,14^. *Fusobacterium nucleatum*, which is found in tumour tissues of colorectal cancer, increases proinflammatory cytokines in colorectal cancer of both rodent models and patients^14^. These studies suggest that cancer microbiota enhances inflammation in the cancer microenvironment.

Similar to heathy human gut microbiota, most cancer microbiotas belong to phyla Proteobacteria, Firmicutes, Actinobacteria, and Bacteroides, which indicates that a major source of cancer microbiota is the gut microbiota^13^. Considering that the liver is anatomically connected to the intestines via the portal vein and that gut bacteria translocate to the liver in chronic liver disease patients, HCC might be also colonised with cancer microbiota. However, the presence of microbiota in liver cancer and its clinical implications remain unclear.

In this study, to investigate whether bacteria are present in liver cancer, especially HCC, we examined microbiota in liver cancer tissues including both primary HCC and metastatic liver cancer. We observed an increase of the diversity in tumour tissues compared with adjacent non-tumour tissues. This trend was evident in patients without HBV or HCV infection. Linear discriminant analysis revealed *Ruminococcus gnavus* as a putative biomarker for viral HCC. This study implies the presence of bacteria in liver cancer tissues and an association with hepatic virus infection.

## Results

### Distinct microbial compositions between tumour and non-tumour regions in the liver

**Figure 1:**
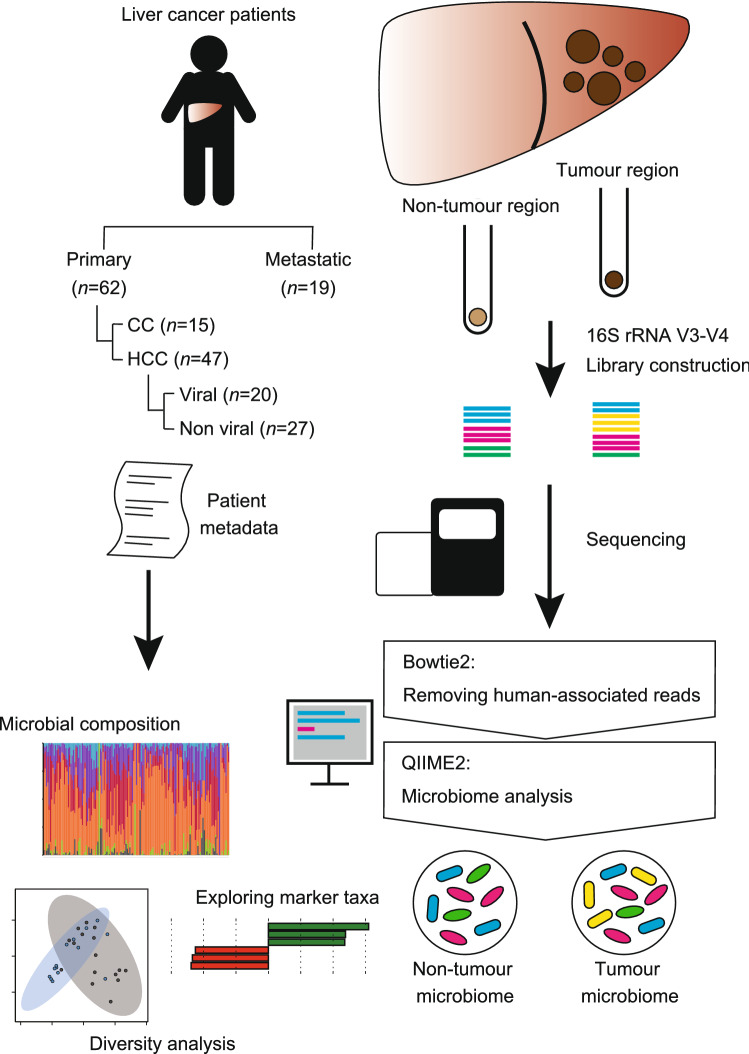
A scheme of the analysis of tumour-associated microbiota in the liver. *CC* cholangiocarcinoma, *HCC* hepatocellular carcinoma.

**Figure 2:**
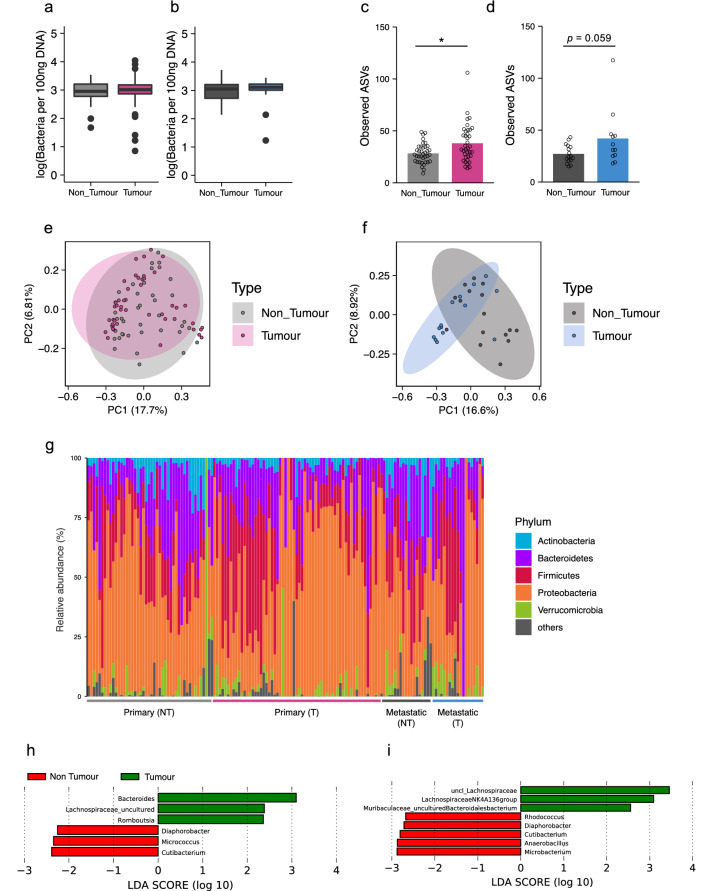
Microbial signature of tumour and non-tumour tissues dissected from primary and metastatic liver cancers. (**a**,**b**) Bacterial load in the non-tumour and tumour regions of primary (**a**) or metastatic liver cancers (**b**). Bacterial load was assessed by qPCR for 16S rRNA gene. The Y axis indicates the number of bacteria as the logarithm (base 10). (**c**,**d**) Species richness (Observed ASVs) of the microbiota in the non-tumour and tumour regions of primary (**c**) or metastatic liver cancers (**d**). (**e**,**f**) Principal component analysis of unweighted UniFrac distances among the microbiota in the non-tumour and tumour regions of primary (**e**) or metastatic liver cancers (**f**). (**g**) The bacterial composition of the microbiota at the phylum level in the non-tumour (NT) and tumour (T) regions of primary and metastatic liver cancers. Each bar represents an individual sample. (**h**,**i**) Discriminating taxa between the non-tumour and tumour regions of primary (**h**) or metastatic liver cancers (**i**) determined by LefSe analysis^15^. The prefix of “uncl_” stands for unclassified bacterium on the SILVA_132 reference database (SSURef_NR99_132_SILVA)^36–38^. Each boxplot represents median, interquartile range (IQR), the lowest and highest values within 1.5 IQRs of the first and third quartiles, and the outliers shown with dots (primary liver cancers: *n* = 37/group; non-tumour regions of metastatic liver cancers: *n* = 12; tumour regions of metastatic liver cancers *n* = 16). Each bar represents mean, and the values of each sample are indicated with dots. Significant differences are indicated at the top of the panels (*p < 0.05; Wilcoxon rank sum test).

To investigate microbial signatures of liver cancers, we analysed tumour and non-tumoural adjacent regions that were surgically resected from patients with primary (i.e., HCC and cholangiocarcinoma) and metastatic liver cancers (Fig. 1). Quantitative PCR (qPCR) analysis revealed no significant differences in bacterial load between tumour regions and adjacent non-tumoural regions both in primary and metastatic liver cancers (Fig. 2a,b). We subsequently performed 16S ribosomal RNA (rRNA) gene sequencing to dissect the bacterial compositions. To eliminate interference by host-derived reads, we filtered human-associated reads from the 16S rRNA sequencing data in advance. For quality control of the analysis, we sequenced PCR amplicons of negative controls (NCs), namely, sterile water or DNA extracts from empty samples. We confirmed that bacterial families in the NCs were only minimally detected in the microbiota of the tumour and non-tumour regions (Supplemental Fig. S1).

We observed that the number of amplicon sequence variants (ASVs) of tumour regions was significantly higher than that of non-tumour regions in primary liver cancers (Fig. 2c). The similar tendency was also observed in metastatic liver cancers, albeit without a significant difference (Fig. 2d). We further assessed beta-diversity by conducting principal component analysis with multiple distance metrics: unweighted- and weighted UniFrac distance, and Bray–Curtis distance. The beta-diversities of tumour regions in primary cancers were also statistically different from those of the non-tumour counterparts in any metric [unweighted UniFrac distance: *p* = 0.006, weighted UniFrac distance: *p* = 0.001, Bray–Curtis distance: *p* = 0.001 (PERMANOVA, number of permutation = 999)] and in metastatic cancer [unweighted UniFrac distance: *p* = 0.002, weighted UniFrac distance: *p* = 0.002, Bray–Curtis distance: *p* = 0.006 (PERMANOVA, number of permutation = 999)] (Fig. 2e,f, and Supplementary Fig. S2a–d).

Taxonomic assignment demonstrated that the hepatic microbiota mainly consisted of bacteria that belonged to Actinobacteria, Bacteroidetes, Firmicutes, and Proteobacteria phyla in patients with both of primary and metastatic liver cancers (Fig. 2g, Supplemental Fig. S1, and Supplemental Data 1). To profile microbes specific for primary or metastatic liver cancers, we analysed the bacterial compositions at the genus level by the linear discriminant analysis effect size (LEfSe) method^15^. We identified *Bacteroides* genus, uncultured bacterium that belonged to Lachnospiraceae family, and *Romboutsia* genus as the feature taxa of the primary liver cancer (Fig. 2h). Moreover, an unclassified genus that belonged to Lachnospiraceae family, Lachnospiraceae NK4A136 group, and uncultured bacterium that belonged to Muribaculaceae family were identified as signature taxa for the metastatic liver cancer (Fig. 2i).

### *Ruminococcus gnavus* as a biomarker for the tumour region in viral HCC patients

**Figure 3:**
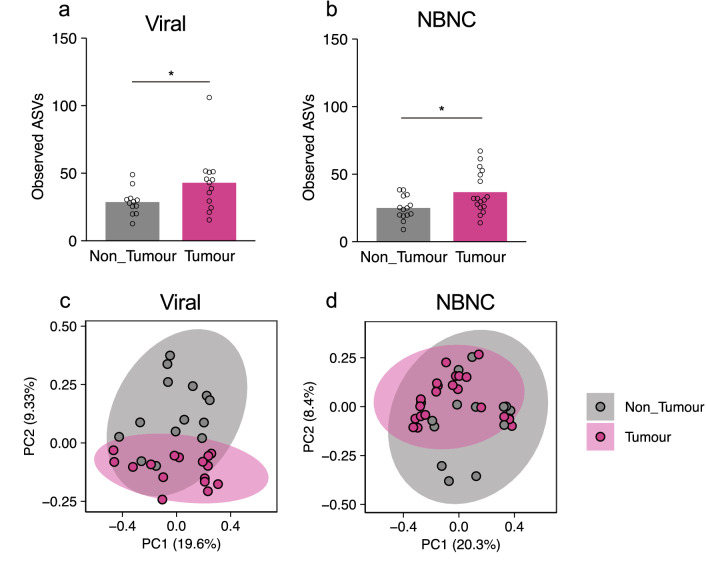
Microbial signature of the non-tumour and tumour regions of viral and NBNC HCC. (**a**,**b**) Species richness (observed ASVs) of the microbiota in the non-tumour and tumour regions of viral (**a**) or NBNC HCC (**b**). (**c**,**d**) Principal component analysis of unweighted UniFrac distances among the microbiota in the non-tumour and tumour regions of viral (**c**) or NBNC HCC (**d**). Each bar represents mean, and the values of each sample are indicated with dots. Significant differences are indicated at the top of the panels (*p < 0.05; Wilcoxon rank sum test).

**Figure 4:**
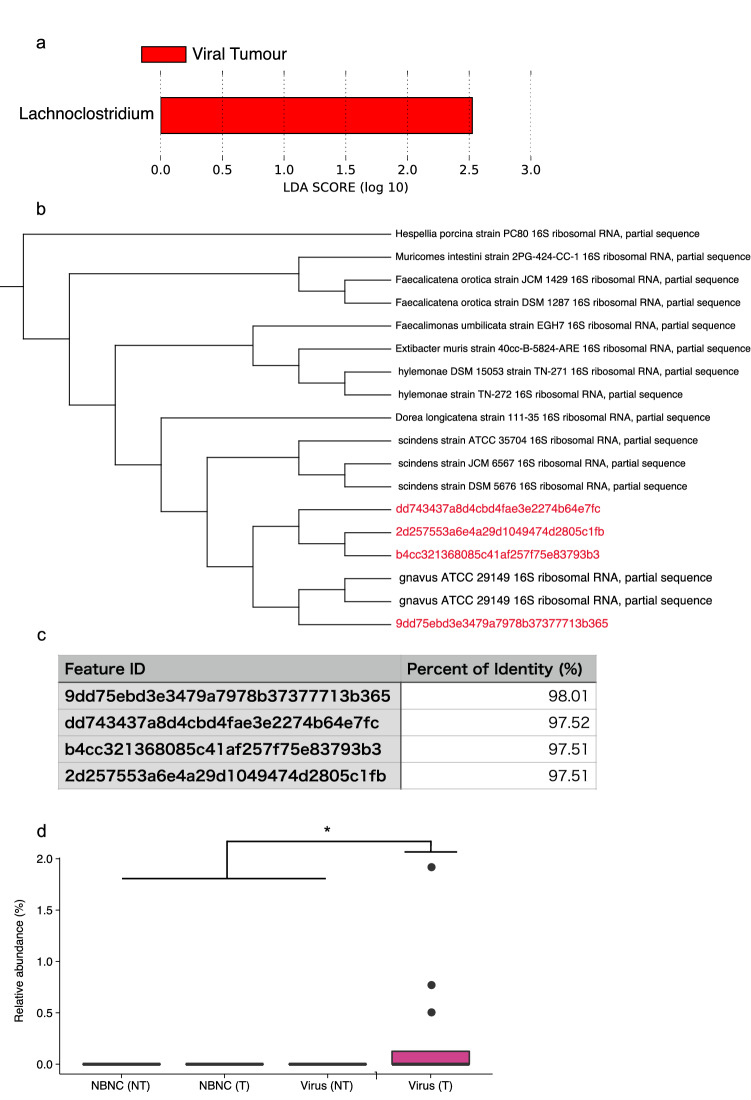
*R. gnavus* is a marker taxon for viral HCC. (**a**) Discriminating taxa between the non-tumour and tumour regions both of viral and NBNC HCC determined by LefSe analysis^15^. (**b**,**c**) Related species of feature sequences assigned as *Lachnoclostridium* genus. Phylogenetic tree based on 16S rRNA gene sequences shows species homologous to the feature sequences (**b**), and the table displays sequence identity of the feature sequences to *R. gnavus* 16S rRNA gene sequence (**c**). (**d**) Relative abundance of *R. gnavus* in the non-tumour (NT) and tumour (T) regions both of viral and NBNC HCC. Each boxplot represents median, interquartile range (IQR), the lowest and highest values within 1.5 IQRs of the first and third quartiles, and the outliers shown with dots. Significant differences are indicated at the top of the panels (*p < 0.05; the Kruskal–Wallis test followed by the pairwise *t* test with Benjamini–Hochberg method). [NBNC (NT): *n* = 18; NBNC (T): *n* = 23; viral (NT): *n* = 15; viral (T): *n* = 12].

Approximately half of the tested HCC patients were infected with HBV and/or HCV (HBV: 13.04%; HCV: 19.57%; Both: 8.70% in HCC samples). We further explored similarities and differences in the tumour-associated microbiota between viral HCC and non-HBV/non-HCV (NBNC) HCC patients. In both viral HCC and NBNC HCC patients, the number of ASVs was significantly greater in the tumour region than in the non-tumour region (Fig. 3a,b). Furthermore, the beta-diversity of microbiota was significantly different between tumour and non-tumour regions in both NBNC HCC [unweighted UniFrac distance: *p* = 0.044, weighted UniFrac distance: *p* = 0.013, Bray–Curtis distance: *p* = 0.028 (PERMANOVA, number of permutations = 999)] and viral HCC [unweighted UniFrac distance: *p* = 0.014, weighted UniFrac distance: *p* = 0.085, Bray–Curtis distance: *p* = 0.005 (PERMANOVA, number of permutations = 999)] patients (Fig. 3c,d, and Supplementary Fig. S3a–d). To identify characteristic taxa of viral or non-viral HCC, we compared the bacterial compositions by the LEfSe method^15^. We detected the *Lachnoclostridium* genus as the only characteristic taxon for viral HCC (Fig. 4a). However, phylogenetic analysis by NCBI MOLE-BLAST revealed that the sequences of the *Lachnoclostridium* genus were homologous to those of *Ruminococcus gnavus* with more than 97% identity (Fig. 4b,c). Occupation of *R. gnavus* was 0.5–2% in viral HCC patients, although this genus was not detected in the non-tumour region of viral HCC patients or in any regions of NBNC HCC (Fig. 4d). Thus, *R. gnavus* is a potential marker taxon to discriminate viral HCC from NBNC HCC.

## Discussion

The tumour-associated microbiotas of primary and metastatic liver cancers largely consisted of Bacteroidetes, Firmicutes, and Proteobacteria whose frequencies were highly diverse among the specimens. This observation could be a common feature of tumour-associated microbiota because these three phyla are also major taxa in various tumour tissues such as bone, breast, colon, lung, and ovarian cancers^13,16–20^. Furthermore, non-gastrointestinal tumour tissues such as lung, ovary, and breast cancers abundantly harbour Actinobacteria, another major phylum of gut microbiota^13,16–20^. However, our data showed that liver tumour-associated microbiota had little, if any, Actinobacteria, similar to the gastrointestinal tumour-associated microbiota^13^. Additionally, we found several marker taxa which are associated with primary or metastatic liver cancers. Among them, we detected *Bacteroides* genera as a marker taxon for the tumour regions of primary liver cancers. The abundance of *Bacteroides* genera is increased in the gut microbiome of patients with NAFLD, non-alcoholic steatohepatitis, and cirrhosis^21–23^. Based on these studies and our data, the overrepresentation of Bacteroides in primary liver cancers might be implicated in the development of liver cancers, although further investigations are necessary to clarify this notion.

Bacteroidetes, Firmicutes, and Proteobacteria detected in the liver cancer-associated microbiota are major members of the gut commensal microbiota in humans^24^. Considering the spatial proximity of the liver to the intestines via the portal vein, liver cancer-associated bacteria are most likely attributed to gut microbiota. In support of this view, dysfunction of the gut epithelial barrier is often observed in patients with chronic liver diseases such as alcoholic hepatitis^25^ and cirrhosis^7,23^, both of which are predisposing factors for liver cancer. Many studies have shown that gut-derived LPS promotes HCC, whereas our data provide evidence that certain bacteria translocate from the gut to the liver. This event may be implicated in the pathology of carcinogenesis, although further investigations are required to verify this notion.

Accumulating evidence has shown that NBNC HCC patients develop intestinal dysbiosis characterised by overrepresentation of proinflammatory bacteria, such as *Escherichia-Shigella* and *Enterococcus*, and underrepresentation of butylate-producing bacteria such as *Faecalibacterium, Ruminococcus*, and *Ruminoclostridium*^26^. Such microbial alterations may contribute to the pathogenesis of NBNC HCC. Similarly, chronic hepatitis patients infected with HBV or HCV also show intestinal dysbiosis. For example, genus *Bacteroides, Veillonella* were overrepresented in the intestinal microbiota of HBV-positive HCC patients, whereas that of HCV-positive HCC patients was characterised by reduction of microbial diversity in association with overrepresentation of genus *Streptococcus* and *Lactobacillus*^27–29^*.* Additionally, we found that *R. gnavus* was associated with the tumour region of viral HCC patients. The abundance of *R. gnavus* increases in patients with inflammatory bowel diseases, especially Crohn’s disease^30–32^. *R. gnavus* produces glucorhamnan that functions as a ligand of TLR4 and eventually induces production of tumor necrosis factor-alpha (TNF-α) from dendritic cells^33^. Because TNF-α promotes hepatocellular carcinogenesis^34^, *R. gnavus* may contribute to the development of viral HCC. Considering that some patients with metastatic liver cancer carry viable bacteria in tumour tissues^35^, investigation of bacterial viability in HCC would facilitate understanding the pathological role of tumour-associated microbiota. However, we acknowledge the limitation of our study, which includes the small sample size for a clinical study, unmatched cohort, and insufficient grouping of NBNC HCC based on habitual data such as alcoholic intake. Therefore, further investigations with larger sample size and matched cohort are needed to establish marker taxa for HCC.

In conclusion, we have characterised tumour-associated microbiota in primary and metastatic liver cancers and identified *R. gnavus* as a marker taxon for viral HCC. Our findings shed light on the microbiome profile of liver cancer, which should facilitate understanding the pathological contribution of tumour-associated microbiota in the liver.

## Methods

### Sample collection

We enrolled 65 patients who underwent hepatectomy from January 2018 to February 2019 at the National Center for Global Health and Medicine (NCGM). Paired liver tumour and adjacent non-tumour tissues were collected from 19 patients with metastatic liver tumours (16 primary colorectal cancer, 2 primary gastric cancers, and 1 primary endometrial cancer). From the other patients with primary liver cancers, 47 hepatocellular carcinoma samples and 15 cholangiocarcinoma samples were collected. These tissue samples were dissected in operating rooms using a sterile surgical knife and tweezers. We renewed a surgical knife and tweezers before dissecting different regions to avoid cross-contamination. The specimens were collected in sterile cryotubes, immediately frozen with liquid nitrogen, and stored at − 80 °C until the analyses. This study was approved by the NCGM research ethics committees (#2464) and Keio University (#190118-1, 190118-2) and informed consent were obtained before sample collection from these patients. All steps were carried out in accordance with national guidelines and regulations.

### DNA extraction and 16S rRNA gene sequencing

Genomic DNA was extracted from liver tissue samples with a DNeasy Blood and Tissue Kit (Qiagen, Hilden, Germany) or QIAamp PowerFecal Pro DNA Kit (Qiagen) in accordance with the manufacturer’s protocols. Before extracting genomic DNA with the DNeasy Blood and Tissue Kit, the tissue samples were homogenised for 10 min with Shake Master Neo (Biomedical Sciences, Tokyo, Japan) and 0.70-mm Garnet PowerBeads (Qiagen). A 16S rRNA genomic library was constructed in accordance with the protocol of the Illumina technical note with some modifications. Briefly, the extracted DNA samples were used as DNA templates in a polymerase chain reaction (PCR) carried out in a reaction mixture of template DNA, KAPA HiFi HotStart Ready Mix, and primers specific for the 16S rRNA V3–V4 region under the following conditions: initial denaturation at 95 °C for 3 min, followed by 35 cycles at 95 °C for 30 s, 55 °C for 30 s, and 72 °C for 30 s. A final elongation step was performed at 72 °C for 5 min. The amplicons were purified with AMPure XP beads (Beckman Coulter, Brea, CA, USA) and attached to dual indices by index PCR with a Nextera XT Index kit (Illumina, San Diego, CA, USA). The libraries were purified with AMPure XP beads (Beckman Coulter). The purified libraries were diluted to 4 nM with Tris–HCl buffer and pooled. The libraries were then sequenced on a Miseq (Illumina) with 300 bp paired-end reads. As negative controls, we sequenced PCR amplicons of negative controls: sterile water or DNA extracts from empty samples.

### Real-time quantitative PCR to estimate bacterial load

Real-time quantitative PCR (qPCR) to estimate bacterial load in liver tissues was performed in accordance with a previously published protocol^11^. Briefly, 2 µl of the extracted DNA was added to a mixture of 5 µl SsoAdvanced Universal SYBR Green Supermix, 2.5 µl sterile water, and 0.5 µl primer mixture containing a forward primer (5ʹ-CCTACGGGNGGCWGCAG-3ʹ) and reverse primer (5ʹ-GACTACHVGGGTATCTAATCC-3ʹ) for the 16S rRNA V3–V4 gene region. The qPCR was performed on a CFX Connect real-time PCR analysis system (Bio-Rad, Tokyo, Japan) at 98 °C for 3 min with 40 cycles at 94 °C for 15 s, 55 °C for 10 s, and 60 °C for 1 min, and completed with melting curve analysis. Each sample was quantified in duplicate. A standard curve was produced with a dilution series (0, 0.05, 0.1, 0.2, 0.8, 3.2, 12.8, and 51.8 pg/µl) of *Escherichia coli* DNA. The standard curve was used to calculate the bacterial number in each sample with the estimation that a bacterium contains 0.005 pg DNA. The Ct values of each sample were compared with that of the standard curve and the bacterial number was calculated as the common log.

### Microbiome analysis

After removing human-associated contaminants using Bowtie2 with the GRch37 index, FASTQ files were analysed using the QIIME2 pipeline (QIIME2 version 2020.2). After conversion to the qza format, the sequence data were demultiplexed and summarised using QIIME2 paired-end-demux. Then, the sequences were trimmed and denoised with the dada2 plugin for QIIME2. Taxonomic assignment was performed using a naïve Bayes fitted classifier trained on the SILVA_132 reference database (SSURef_NR99_132_SILVA) with the feature-classifier plugin for QIIME2^36–38^. The phylogenetic tree for diversity analysis was reconstructed using QIIME2 align-to-tree-mafft-fasttree. Diversity analysis ware performed with QIIME2 core-metrics-phylogenetic. Relative abundances of each taxon were calculated using the taxa collapse QIIME2 plugin.

### Statistics

To analyse the number of observed ASVs, bacterial load, and the abundance of each bacterium, statistical differences were examined by the Student’s *t* test for comparisons between two groups or Tukey’s multiple comparison test to compare multiple groups in the case of homogenous variance. In the case of heterogeneous variance, statistical differences were examined by the Wilcoxon rank-sum test for two groups or the Kruskal–Wallis test followed by the pairwise *t* test with Benjamini–Hochberg method. Identification of feature taxa was performed by the LEfSe method^15^. Statistical analyses with the exception of LefSe analysis and visualisation were performed with R version 4.0.2.

## Supplementary Information

Supplementary Figures.

Supplementary Information.

## Data Availability

Datasets from DDBJ (accession# DRA 011436) are publicly available. The datasets generated during and/or analyzed during the current study are available from the corresponding author on reasonable request.
