# Supplementary material for: Profiling of tumour-associated microbiota in human hepatocellular carcinoma
Source: Sci Rep. 2021 May 19;11:10589. doi: 10.1038/s41598-021-89963-1 (PMC8134445; doi:10.1038/s41598-021-89963-1)
Supplement: Supplementary file 1 — Supplementary Figures. [file 41598_2021_89963_MOESM1_ESM.pdf]

Supplementary Information

Profiling of tumour-associated microbiota in human hepatocellular carcinoma

Seiga Komiyama, Takahiro Yamada, Nobuyuki Takemura, Norihiro Kokudo, Koji Hase, Yuki I. Kawamura

- Supplemental Figures S1-S3

Supplementary Figure S1

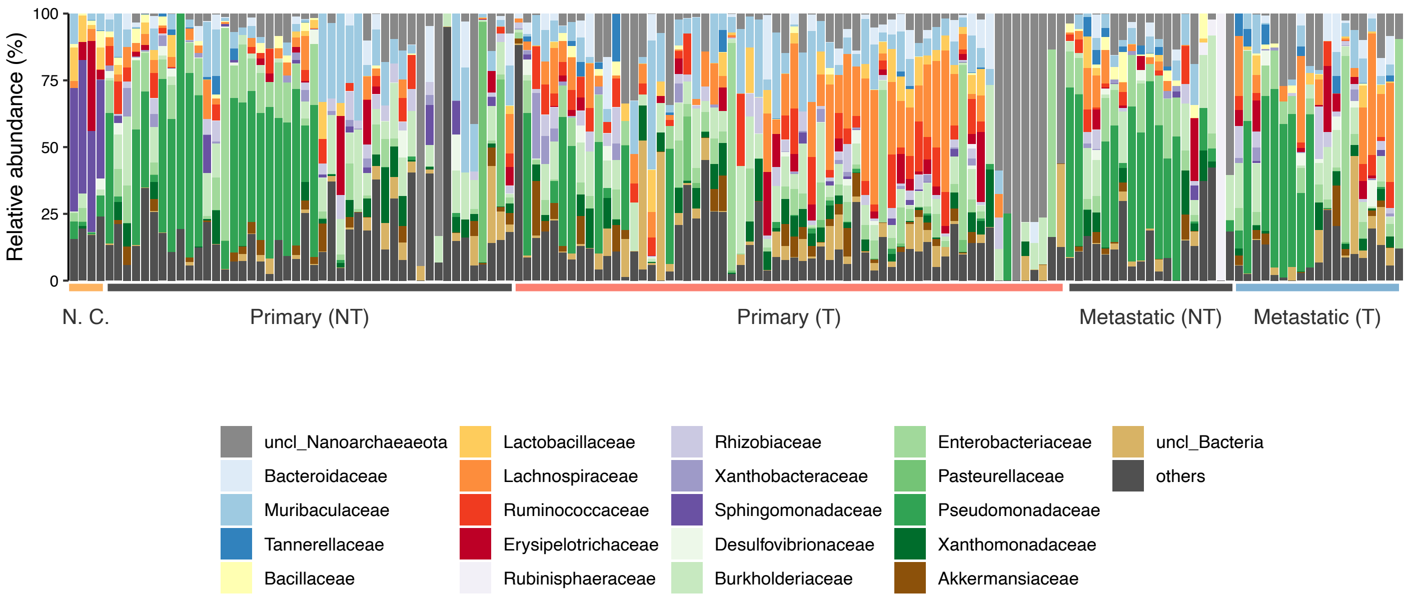

Supplementary Fig. S1. The microbial composition of the tumour and non-tumour regions of primary or metastatic liver cancers.

The microbial composition of the negative controls (N. C.), and the tumour (T) and non-tumour (NT) regions of primary or metastatic liver cancers.

Supplementary Figure S2

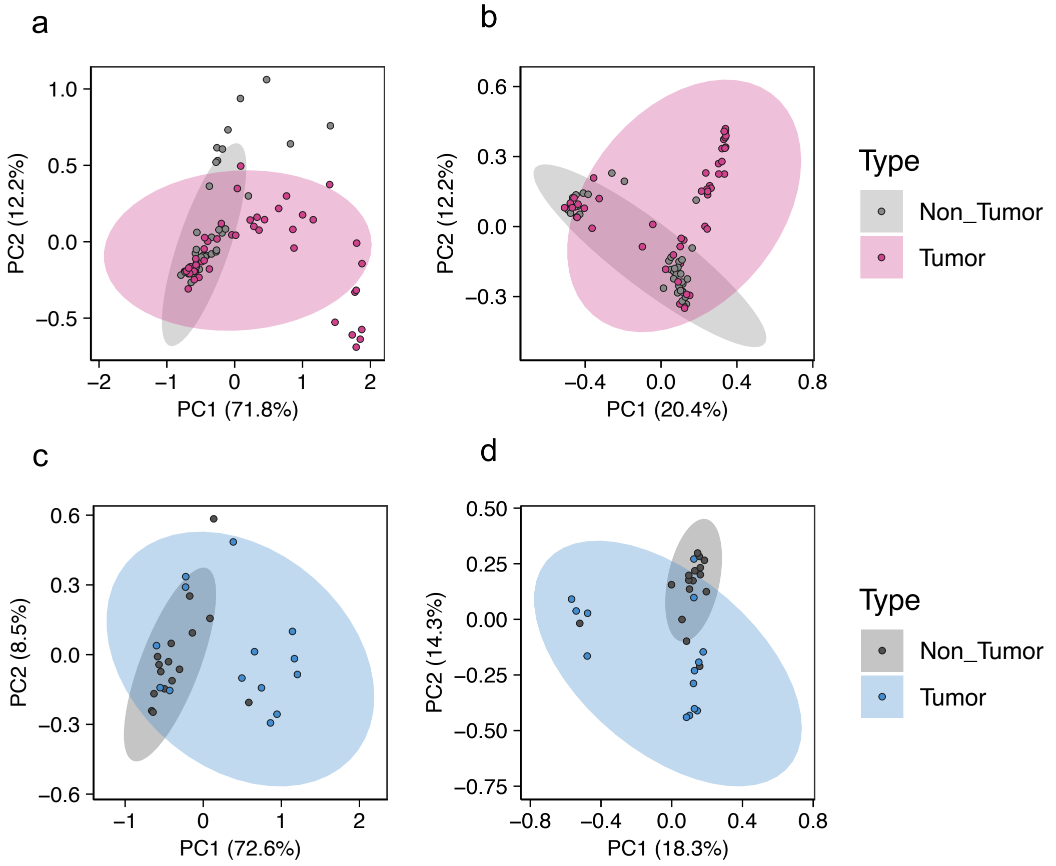

Supplementary Fig. S2. The beta diversity of the microbiota in the tumor and non-tumor regions of primary or metastatic liver cancers.
(a-d) Principal component analysis of weighted UniFrac distances (a, c) or Bray-Curtis distances (b, d) among the microbiota in the non-tumor and tumor regions of primary (a, b) or metastatic liver cancers (c, d).

Supplementary Figure S3

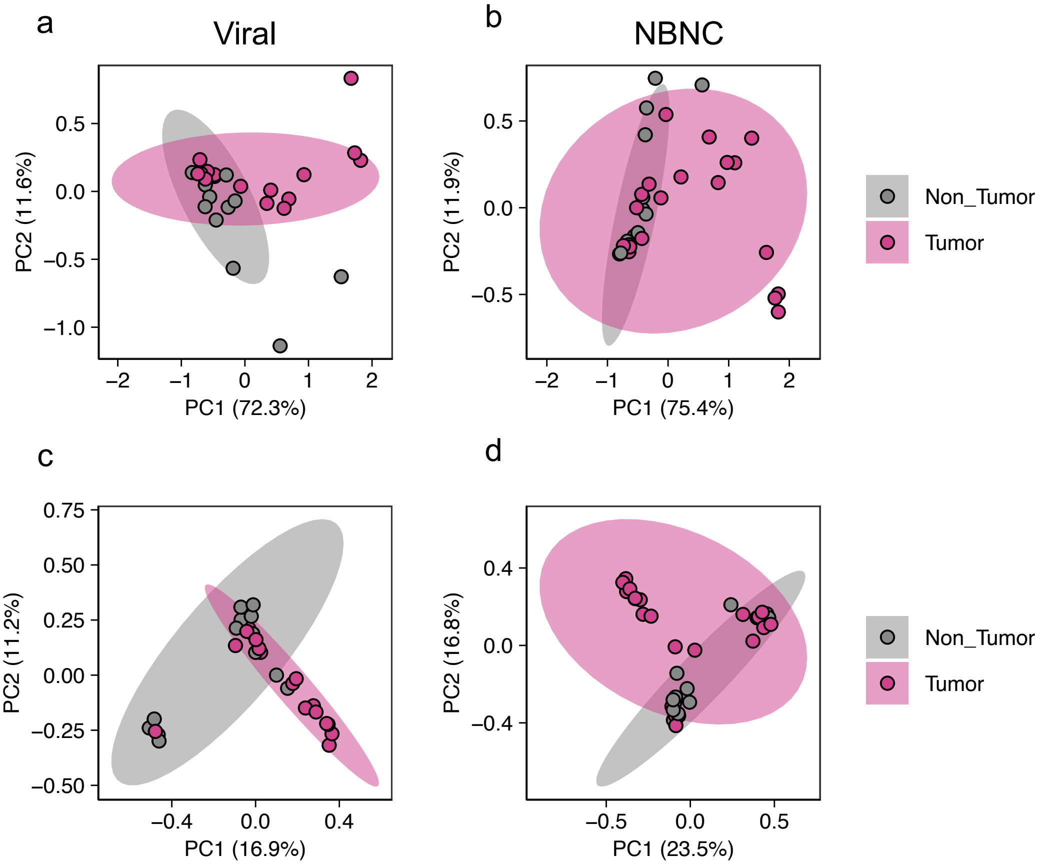

Supplementary Fig. S3. The beta diversity of the microbiota in the tumor and non-tumor tissues of viral or NBNC HCC.
(a-d) Principal component analysis of weighted UniFrac distances (a, b) or Bray-Curtis distances (c, d) among the microbiota in the non-tumor and tumor regions of viral (a, c) or NBNC HCC (b, d).
